# Temporal analysis of mRNA expression profiles in *Orientia* infected C3HeB/FeJ mouse

**DOI:** 10.1186/s12866-019-1684-3

**Published:** 2020-01-06

**Authors:** Chien-Chung Chao, Ruoting Yang, Zhiwen Zhang, Tatyana Belinskaya, Chye-Teik Chan, Stacy-Ann Miller, Rasha Hammamieh, Marti Jett, Wei-Mei Ching

**Affiliations:** 1 Viral and Rickettsial Diseases Department, Infectious Diseases Directorate, Naval Medical Research Center, 503 Robert Grant Ave, RM3N71, Silver Spring, MD 20910 USA; 2 Uniformed Services University of the Health Sciences, Bethesda, MD USA; 3 Integrative Systems Biology, US Army Medical Research and Material Command, USACEHR, Ft Detrick, Ft Federick, MD USA; 4 Advanced Biomedical Computation Science, Frederick National Laboratory for Cancer Research, Frederick, MD USA

**Keywords:** Scrub typhus, cDNA microarray, *Orientia tsutsugamushi* infection, Semi-quantitative PCR, Gene expression profiles

## Abstract

**Background:**

Scrub typhus causes up to 35% mortality if left untreated. One billion people living in the endemic regions are at risk. In spite of its heavy disease burden in some of the most populated areas in the world, there is no vaccine available. Although the disease can be effectively treated by proper antibiotics, timely and accurate diagnosis remains a challenge. *Orientia tsutsugamushi* infects a variety of mammalian cells in vitro and replicates in the cytoplasm of the infected cells. Microarray analysis has been used extensively to study host-pathogen interactions in in vitro models to understand pathogenesis. However there is a lack of in vivo studies.

**Results:**

In this study, C3HeB/FeJ (C3H) mice were infected by *O. tsutsugamushi* via the intraperitoneal route and monitored gene expression at 10 different time points post infection. We observed two distinct types of expression profiles in the genes that we analyzed. There are two valleys (4–18 h and 2–4 days) with low number of differentially expressed genes (DEG) with three peaks with high number of DEG at 2 h, 1-day and 7-day post infection. Further analysis revealed that pathways like complement and coagulation cascade, and blood clotting cascade pathways showed significant global changes throughout entire time course. Real time quantitative Polymerase Chain Reaction (RT-qPCR) confirmed the change of expression for genes involved in complement and coagulation cascade. These results suggested dynamic regulation of the complement and coagulation cascades throughout most of the time post infection while some other specific pathways, such as fatty acid metabolism and tryptophan metabolism, are turned on or off at certain times post infection.

**Conclusions:**

The findings highlight the complex interconnection among all different biological pathways. It is conceivable that specific pathways such as cell growth control and cell development in the host are affected by *Orientia* in the initial phase of infection for *Orientia* to grow intracellularly. Once *Orientia* is replicating successfully inside the host as infection progresses, the infection could activate pathways involved in cellular immune responses to defend for host cell survival and try to eliminate the pathogen.

## Background

Scrub typhus is a febrile illness caused by an obligate intracellular bacterium, *Orientia tsutaugamushi*. The disease is transmitted by the bite of the larvae of various species of trombiculid mites. Humans are the accidental hosts while the natural hosts for these mites are rodents. Scrub typhus is endemic in Asia-Pacific region and up to 23% of all febrile illnesses can be attributed to scrub typhus [1, 2]. Scrub typhus is endemic within a “tsutsugamushi” triangle area that is about 13 million square kilometers which includes Pakistan, India and Nepal to the west, Japan to the east, southeastern Siberia, China, and Korea to the north and Indonesia, Philippines, northern Australia and the intervening Pacific islands to the south [3–12]. This area is one of the most populated areas and an estimated 1 billion people living in this area are at risk. Recent evidence has suggested that the disease is expanding beyond the traditional Asia-Pacific area to include the Middle East, South America and Africa [2, 13–15]. The disease is characterized by fever, rash, eschar, pneumonitis, meningitis, and in some severe cases, patients can have disseminated intravascular coagulation that may lead to circulatory failure [16]. In most scrub typhus patients, the symptoms are relatively mild, a timely, accurate and differential diagnosis can be challenging [17]. Therefore, although the disease can be effectively treated by doxycycline, oftentimes the patients are left untreated due to the lack of accurate diagnosis. The mortality in this untreated population can be as high as 35% [1, 17]. Furthermore, cases of ineffective doxycycline treatment have been documented in Thailand and India, suggesting the potential rise of antibiotic resistant strains of *O. tsutsugamushi* in endemic areas [18, 19].

*Orientia* is extremely labile and can infect a variety of mammalian cells ex vivo, including endothelial cells [20], dendritic cells [21], monocytes [21] and polymorphonuclear leukocytes [22]. Once they are in mammalian cells, it is believed to replicate in the cytoplasm of the infected cells. This is supported by previous studies showing that *O. tsutsugamushi* induces phagocytosis of host cells and then escapes from the phagosome in 30 min by lysing the phagosomal membrane [23]. Nevertheless, the exact mechanisms by which *O. tsutsugamushi* enter the host cells and escape from the host endocytic pathway have not yet been elucidated. Human endothelial cells are believed to be the target cells mainly because the results of immunohistochemistry using autopsy tissues of scrub typhus patients [24]. In contrast to autopsy results, dendritic cells and monocytes rather than endothelial cells are the target cells in eschars of scrub typhus patients [21], suggesting that the cells first encounter (i.e., dendritic cells and monocytes) *O. tsutsugamushi* can be different from those cells where *O. tsutsugamushi* are subsequently disseminated (endothelium) and accumulated. The involvement of local host immune responses and the mechanism for following systemic dissemination of *Orientia* may be very important to elucidate infection mechanism. A lot of efforts have focused on the immune responses upon infection of *O. tsutsugamushi* in various animal models to better understand the pathological observations in patients. Traditionally, the intraperitoneal (IP) inoculation is the route of choice to infect mice and has been used to evaluate the efficacy of several vaccine candidates [25–28]. Other routes for inoculation, such as intradermal (ID) [29, 30], intravenous (IV) [31–34], and more recently footpad [35], have been used in mouse models to study immunological responses. From all these studies, it appears that parameters like variations in route of inoculation, the mouse strains used, and strains of *O. tsutsugamushi* inoculum, can often lead to different challenge outcomes (e.g., lethal vs. non-lethal challenge models). However, it is difficult to conclude whether certain route or strain of mouse or strain of *O. tsutsugamushi* inoculum is really superior to the other as these studies had different emphases. Some models observed whether mice succumb to *O. tsutsugamushi* infection to evaluate the efficacy of vaccine candidates (i.e., IP), others suggested that a good mouse model is the one that shows similar pathology and target cells as observed in human scrub typhus (i.e., ID), and still others suggested that a good model should use an inoculation route that is similar to the natural infection route (i.e., ID or footpad). In addition to mouse models, a non-human primate monkey model has recently been used to evaluate the leading vaccine candidates [36, 37]. The study of the immune responses in non-human primate model is not as comprehensive as that in mouse model.

Microarray technology has been used extensively to investigate the host transcriptomic responses in various infectious diseases. Results from experiments conducted ex vivo or in mouse model have revealed insights into mechanisms of pathogenesis [38–41]. Similarly, microarray technology has been used to study host responses upon infections in animal models [42–45].

In this study, we inoculated C3H mice with *O. tsutsugamushi* via the IP route and characterized the changes in gene expression profiles at 10 different time points post inoculation. We observed two valleys (4–18 h and 2–4 days) of low number of differential expressed genes (DEG) with three peaks of high number of DEG at 2 h, 1 day and 7 days post inoculation. A spike pathway network analysis was performed and showed, in addition to a more general change of expression in pathways like the complement and coagulation cascades throughout most of the time course, there were also pathways that appear to be turning on or off at certain times post-inoculation. Further RT-qPCR was performed to confirm the expression of genes involved in complement and coagulation cascade. The findings highlight the complex connection of all different biological pathways. It is conceivable that during the initial phase of infection, *Orientia* is actively affecting pathways such as cell growth control, extracellular matrix adhesion and cell development in the host in order to survive. At later time post-infection, genes involved in cellular immune responses are triggered to defend infection and try to eliminate the pathogen.

## Results

### Clinical observation

Five mice per time point were observed for apparent clinical symptoms. All mice appeared healthy during the first 9 days with no signs of disease or illness. There was no eschar formation or any observable reaction at the injection site in any of the mice by 9 days post inoculation (dpi). Ruffled fur and loss of mobility were observed starting 9 dpi. All the 5 mice monitored for survival succumbed to the infection by 12 dpi, with an average survival of 11.2 dpi.

### Antibody response following inoculation

Serum samples from each mouse were used to monitor the antibody responses elicited by *O. tsutsugamushi* infection. No *Orientia*-specific antibodies were detected in infected mice before or at 7 dpi. At 10 dpi, two mice were found non-responsive to outside stimuli and were euthanized to collect blood and tissues. The sera from these two mice had detectable IgG and IgM antibodies against the *Orientia* 56 kDa immunodominant protein, but the antibodies against the 47 kDa protein antigen were still undetectable. Antibodies were detected by 10 dpi, indicating that antibodies rose to detectable levels between 7 dpi and 10 dpi. This observation coincided with the appearance of ruffled fur and loss of mobility.

### Detection of circulating soluble cell adhesion molecules

**Fig. 1:**
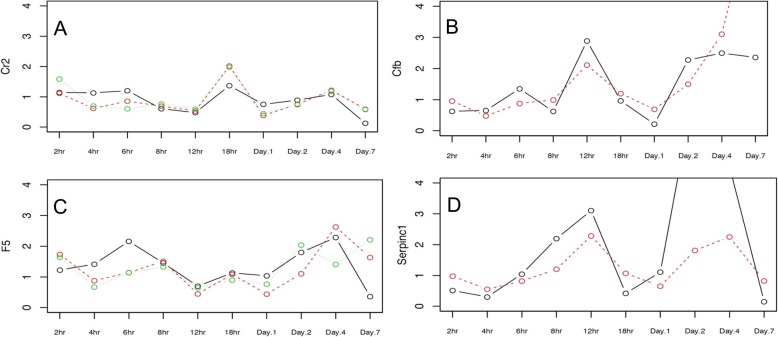
Effect of infection on the release of ICAM (**a**), L-Selectin (**b**) and VCAM (**c**). Sera from sham inoculated (open bars) and *Orientia* challenged (black bars) mice were collected from selected time points as indicated. ELISA was used to quantitate the amount of each protein in serum samples using a standard curve established per manufacturer’s instruction. * Significantly different was determined by student’s *t*-test (*p* < 0.025)

**Fig. 2:**
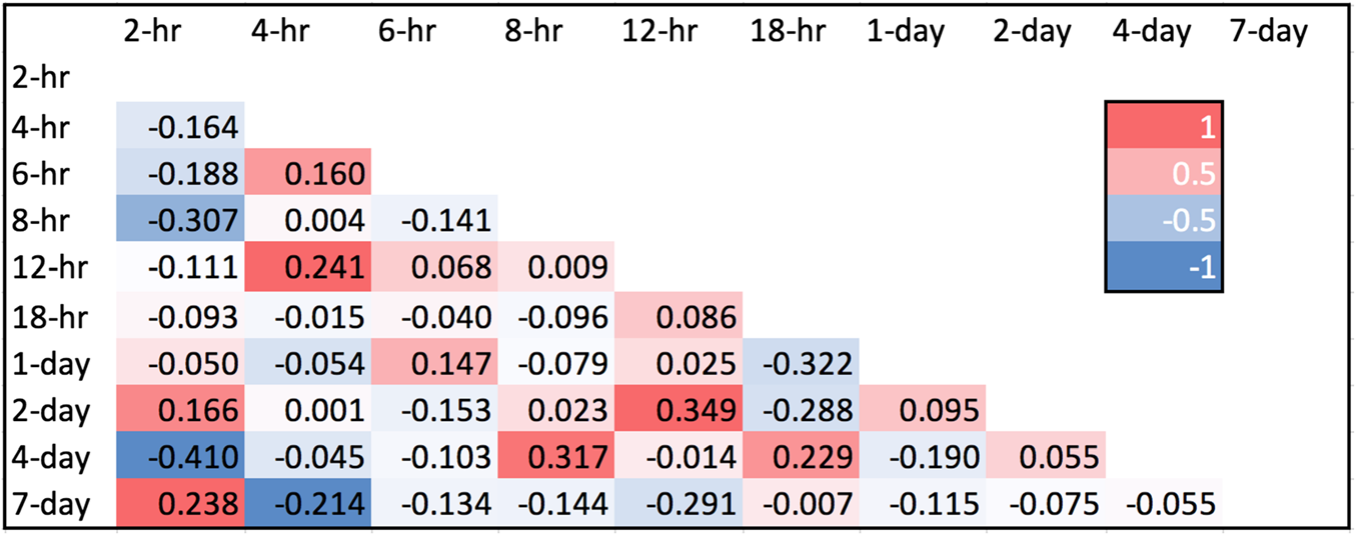
Correlation matrix of the fold changes at each time point**.** Clear distinction of the fold change was observed at 2-h vs. 8-h, 2-h vs 4-day, 8-h vs 2-day and 12-h vs 2-day

In order to investigate surrogate markers of endothelial and leukocyte activation following IP inoculation of *O. tsutsugamushi*, serum levels of circulating L-selectin, ICAM-1, and VCAM-1 were measured at selected time points. As shown in Fig. 1a, the amount of ICAM decreased as early as 8-h post inoculation (hpi) and continued to be lower than sham samples until 7 dpi when infected samples had more ICAM. Similar patterns were found for L-Selectin (Fig. 1b). The trend for VCAM (Fig. 1c) was different. The infected samples started to show increased amounts as early as 1 dpi and were higher than sham samples from 1 dpi to 7 dpi. Statistically significant differences in the amounts of these cell adhesion molecules between sham and infected samples were observed with ICAM at 8 hpi and 1 dpi. (Fig. 2a), for L-Selectin at 4 dpi and 7 dpi. (Fig. 2b), and for VCAM at 7 dpi. (Fig. 2c).

### Tissue tropism of *O. tsutsugamushi* in IP inoculated mice

To determine the distribution of *O. tsutsugamushi* in mouse tissues after IP inoculation, lung, liver, and spleen were collected for bacterial quantitation by the *Orientia*-specific 47 kDa qPCR assay. Negligible amounts of *O. tsutsugamushi* were detected in all three tissues at 4 dpi (Table 1). Appreciable amounts of *Orientia* DNA were detected at 7 dpi and 10 dpi. Among these tissues, the liver had the highest amount of *O. tsutsugamushi* at all time points (4 dpi, 7 dpi, and 10 dpi), followed by the spleen, and then the lung. The fold increase of *Orientia* between 4 dpi and 7 dpi was similar to that between 7 dpi and 10 dpi for both liver and lung, leading to very high *Orientia* load in these tissues. The fold increase was much more moderate for the spleen.

**Table 1:** Dissemination of *O. tsutsugamushi* in mouse tissues^a^

# of 47 kDa gene/10^5^ mouse cfd gene (fold increase)^b^			
Time post infection	Liver	Spleen	Lung
< day 2	6.8 + 7.5^c^	ND^d^	0.8 + 0.4^e^
Day 4	6.6 + 1.6 (0.97)	6.3 + 3.9 (n/a)^g^	1.5 + 0.9 (1.9)
Day 7	1473 + 488 (223)	290 + 224 (46.0)	244 + 92 (163)
Day 10^f^	3.9 × 10^5^ + 1.8 × 10^5^ (264.7)	8701 + 4370 (30.0)	52.4 × 10^3^ + 7.1 × 10^3^ (214.9)

^a^Numbers represent average of three independent qPCR quantitations of mouse and *O. tsutsugamushi* copy numbers using cfd and 47 kDa gene, respectively, as described in materials and methods. The number of *O. tsutsugamushi* 47 kDa gene/10^5^ cfd gene is reported as mean + standard deviation

^b^Numbers in parentheses represent fold increase over previous time point

^c^Average of detectable *O. tsutsugamushi* 47 kDa gene from 4 hpi, 6 hpi, and 18 hpi samples. Samples from other time points before 2 dpi did not have detectable 47 kDa gene

^d^ND: Not determined

^e^Average of detectable *O. tsutsugamushi* 47 kDa gene from 12 hpi samples. Samples from other time points did not have detectable 47 kDa gene

^f^Only two mice had tissues collected to perform qPCR

^g^Not applicable

### Correlation of fold change

We applied Pearson’s test to correlate the fold change of expression at each time point. The higher the correlation indicates the higher similarity in gene responses between the two time points (Fig. 2). Noticeably, the gene response at 2 hpi was negatively correlated to 8 hpi (− 0.307) and 4 dpi (− 0.410), suggesting the expression between 2 hpi was opposite to that of 8 hpi and 4 dpi. In contrast, 12 hpi vs 2 dpi (0.349) and 8 hpi vs 4 dpi (0.317) have positive correlations, indicating that the gene responses between these time points are sharing similar patterns.

### Individual differentially expressed probes analysis

**Fig. 3:**
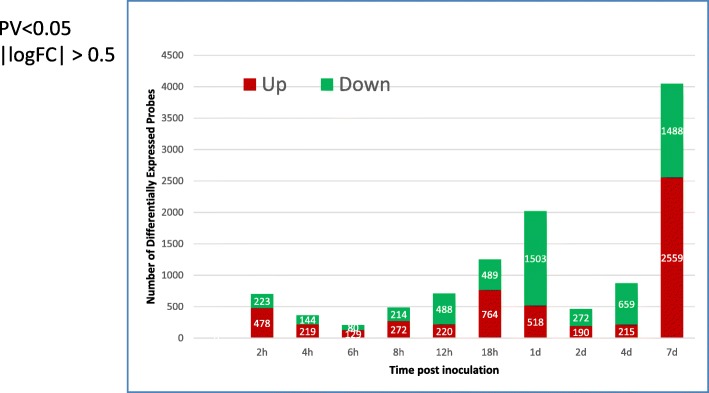
A three-phase response of differential expression of genes. The number of differential expressed genes (Y-axis) at different time post inoculation (X-axis) was plotted showing a two-phase response. One initial phase at 2 hpi followed by an early phase between 4 hpi to 1 dpi and a late phase between 2 dpi to 7 dpi. The number of up-regulated genes is shown in red bars and the number of down-regulated genes is shown in green bars

A rank-sum test was used to identify the Differentially Expressed Probes (DEPs) (defined by *p*-value< 0.05 and |log(fold change)| > 0.5) for individual time points. We also applied moderated *t*-test (limma, R package). The number of DEPs from *t*-test is generally 1.5 times more than that of the rank-sum test, but the trend is the same. The stacked bar chart of Fig. 3 highlights the number of up-regulated (red) and down-regulated (green) DEPs. Significantly larger numbers of DEPs were observed at 2 hpi, 1 dpi, and 7 dpi than those of the adjacent times. After an initial response to IP inoculation at 2 hpi, it seemed that at 6 hpi and 8 hpi. Fewer probes showed significant changes, while from 8 hpi there is a sharp jump in DEPs (~ 2000 DEPs). However, it suddenly dropped to ~ 400 DEPs at 2 dpi and then gradually increased to more than 800 DEPs. Therefore, the three peaks at 2 hpi, 1 dpi, and 7 dpi may suggest a three-phase response, with an initial response to infection (i.e. 2 hpi) then 2 cycles (i.e., early (4 hpi – 1 dpi), and late stage (2 dpi – 7 dpi)) of increasing number of significantly expressed genes. The initial phase at 2 hpi might be different from the later two phases as they took longer to develop.

### Gene cluster analysis

**Fig. 4:**
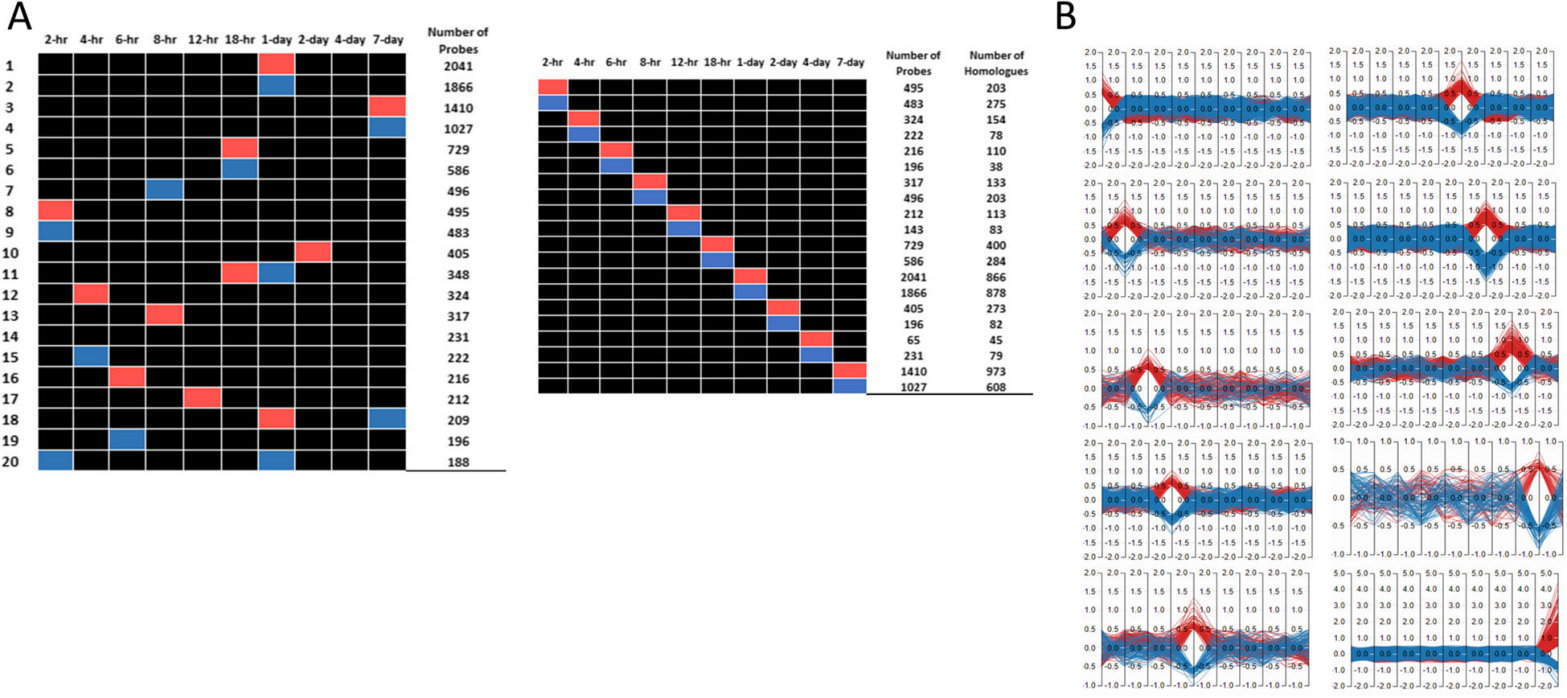
Spike pattern analysis of top 20 patterns with most significantly up- or down-regulated probes. Genes that are showing significant up- or down-regulation between infected and control mice were clustered together based on time post-inoculation. Panel **a**: Top 20 clusters (#1- #20, left Y-axis)) were identified by Functional analysis Heatmap. These clusters were ranked in descending order based on the number of probes (e.g., 2041, right Y-axis) determined as significantly up- or down-regulated at different time post-inoculation (X-axis). The assignment of “0” (gray), “-” (blue), and “+” (red) to certain expression pattern was defined as described in **Methods**. It is noted that these top 20 clusters were mostly single time point up- or down-regulated in the infected group in comparison to the sham group. Panel **b**: Number of probes showing up-regulation (red) and down-regulation (blue) at different time post-infection

**Fig. 5:**
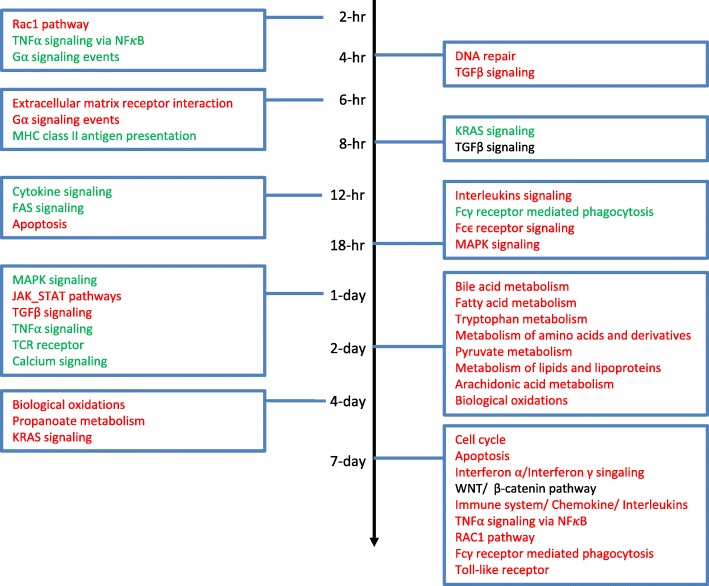
Pathway enrichment at each time point. Specific pathways that showed significant regulation (red: pathways with more up-regulated genes, green: pathways with more down-regulated genes, and black: pathways with equal up- and down-regulated genes) in response to *Orientia* infection at different time points are shown. There is no single pathway showing particular enrichment at all time points. The pathways listed are just a fraction of many pathways that are enriched

We further dissected the DEGs into spike patterns based on number of significantly up- or down-regulated probes at different time points. Figure 4 panel A shows top 20 spike patterns with the number of up- and down-regulated probes in descending order. These results revealed that three different spike patterns showed the highest number of probes with significant changes occurred at 2 hpi (pattern #8, #9 and #20), 18 hpi (pattern #5, #6 and #11), and 7 dpi (pattern #3, #4 and #18). On 1 dpi, 5 different patterns showed most up- and down-regulated genes. The majority of these 20 spike patterns had only single time point with most significantly up- and down-regulated probes. The exceptions were pattern #11, #18 and #20. Figure 4 panel B shows the number of probes /genes that are significantly up- or down-regulated at each time point. We further studied the enriched pathways of the top 20 clusters. As shown in Fig. 5, Rac1 pathway (up), TNFα signaling via NFκB (down), TGFβ signaling (down), interleukins signaling (up), and cytokine signaling (down) pathways are in general the major enriched pathways from 2 hpi to 1 dpi. Various signaling pathways were commonly expressed from 18 hpi, 1 dpi, and 7 dpi. Many metabolic pathways emerged at 2 dpi but disappeared later. Inflammation signaling pathways were activated between 18 hpi and 1 dpi. Various metabolic pathways were up regulated at 2 dpi and 4 dpi. At 7 dpi many pathways encompassing various biological functions such as WNT, TNF, mTOR, interferon, cytokines/chemokines, and toll-like receptor pathways were shown to be significantly up-regulated. The detailed enriched functions are listed in Additional file 1: Table S1.

### Global pathway analysis

**Fig. 6:**
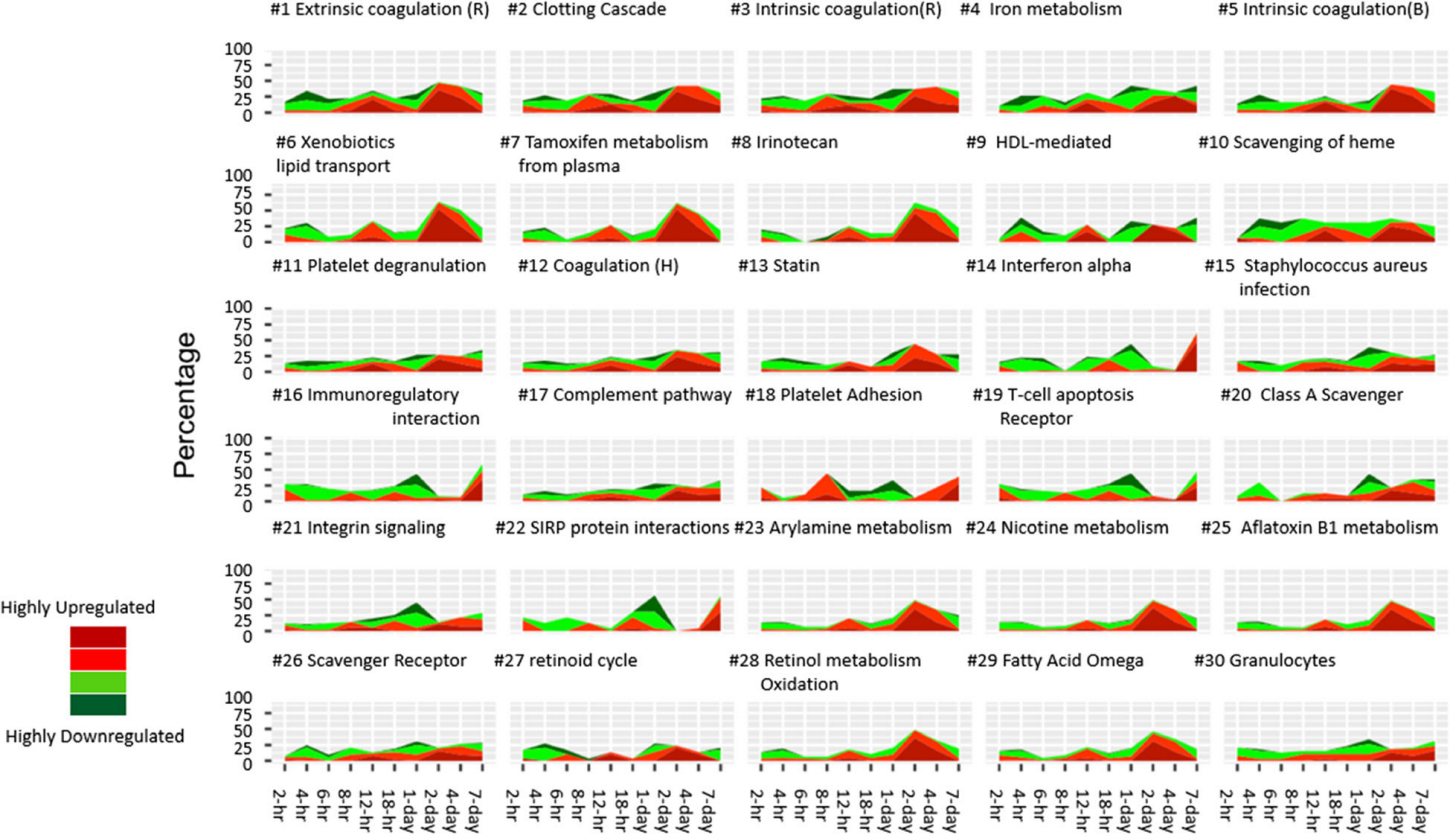
Top 30 pathways with most significant changes. Pathway analysis was performed on expression of all genes involved in various pathways. The top 30 pathways with most genes showing significant regulations throughout the entire time course were ranked. Among all these pathways, as much as 40% of all genes involved in specified pathways were up or down regulated throughout the entire time course. The dark green, green, red, and dark red stand for the percentage of the genes with log2 fold change of (−∞, − 1), (− 1,-0.5), (0.5, 1), and (1, +∞), respectively

Pathway enrichment analysis identified the top 30 pathways with significant changes throughout the entire time course (Fig. 6). In general, around 40% of all genes involved in these pathways were significantly regulated. Several clusters pointed to pathways related to platelet degranulation and adhesion, coagulation, immune system (interferon alpha and immune-regulatory interaction), complement pathway, and iron metabolism.

### Confirmation of selected pathways using qRT-PCR

**Fig. 7:**
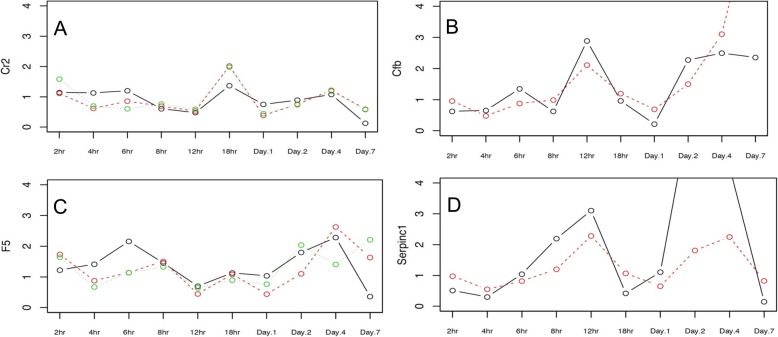
Representative qRT-PCR confirmation of genes involved in complement (**a** and **b**) and coagulation (**c** and **d**) pathways. The qRT-PCR was performed as described in **Methods**. The fold change determined by microarray (dotted lines) and qRT-PCR (solid lines) showed good correlation

The differential expression profiles of genes involved in blood coagulation cascades and classic complement pathways were confirmed using qRT-PCR. The fold-change of gene expression resulting after *Orientia* infection was confirmed. Four genes, including genes involved in complement pathway (C2 and cfb) and coagulation pathways (F5 and SerpinC1), were shown in Fig. 7 as examples to demonstrate that similar trends were observed by microarray probes (dotted lines) and qRT-PCR (solid lines).

## Discussion

Significant variations of gene expression in many key biological pathways are observed in C3H mice infected by *Orientia*. The C3H mouse model is a lethal model and all mice that received the 250 mLD_50_ of *Orientia* were sick and succumbed to the infection within 21 dpi. The expression profiles, while varying significantly for specific pathways, can be grouped into two valleys (4–18 hpi and 2–4 dpi) of differentially expressed genes and three peaks of differentially expressed genes at 2 hpi, 1 dpi, and 7 dpi. There were no genes associated with certain pathways showing a stable and continuous up- or down-regulation throughout the entire time course.

The IP challenge route resulted in dissemination of *Orientia* to different tissues in a time-dependent fashion with the maximum copy number of *Orientia* detected in the liver. Other challenge routes have shown different tissue tropisms, suggesting that different challenge routes may lead to different immune responses [29–35]. *Orientia* was detected in the liver as early as 7dpi, which is slightly earlier than the visible clinical signs, i.e., ruffled fur. It is intriguing that the lung, which is one of the target tissues associated with severe ST, appeared to have accumulated more *Orientia* in comparison to the spleen. We have also observed that higher numbers of *Orientia* accumulated in the lung were associated with delayed recovery in mice partially protected by immunization using recombinant protein antigens (data not shown). Leukocytes and endothelial cells were activated based on the level of surrogate markers. All three of these markers showed highest levels in infected mice at 7 dpi with a general upward trend suggesting that the activation occurs at around the same time when signs of sickness appeared and dissemination of *Orientia* to different tissues were detected. Both IL-1 and TNFα pathways were significantly up-regulated at 7 dpi and this may contribute to the upward trend of the three markers as the infection progressed.

There are pathways in which 40% of all genes involved were significantly regulated throughout the entire time course. Among these pathways, the complement, platelet and coagulation pathways are particularly interesting as they are among the top 30 pathways. These include intrinsic and extrinsic coagulation pathways, complement pathway, and platelet degranulation and adhesion pathways. While these 7 pathways have their respective unique pathophysiological roles, they do share some commonalities, particularly with respect to their innate defences against external threats (i.e., *Orientia* invasion) [46]. There are several interesting observations to support our results. First, it was observed in an ectoparasite infection fish model that a non-lethal dosage of infection resulted in significant down-regulation of many genes in these pathways [47] similar to what we observed in the early phase of infection. On the contrary, when a lethal dose of infection was administered in the fish model, many of these same genes were up-regulated, just as what was observed in the late phase of *Orientia* infection. Second, our results are similar to a genome-wide transcriptome profiling of a murine acute melioidosis model in which early activation of complement system was observed at 24 h post-infection to maintain host cellular homeostasis and eliminate intracellular bacteria [48]. Furthermore, a recent prospective study from Laos has shown that markers of coagulation activation and all inflammatory cytokines are significantly elevated, and in vivo coagulation activation ears prominent and seems to be related to a strong proinflammatory response [49] in scrub typhus pappatients. Finally, the activation of the complement system can also directly induce platelet activation and aggregation [50], and trigger the induction of tissue factor expression and activity in endothelial cells and neutrophils. The activation of endothelial cells may explain the procoagulant properties in scrub typhus patients with acute respiratory distress syndrome [51–53].

Among the 20 clusters of gene expression patterns, unique and shared pathway enrichments were observed at different time points. Especially noteworthy pathways in the early phase (2 hpi) of infection included Rac1, TNFα signaling via NFκB and G_α signaling events. These genes are mainly responsible for cell growth control, cytokine secretion, cell motility, extracellular matrix adhesion, cell transformation and invasion, and cell development. In the case of TNFα signalling via NFκB, these genes signify that NFκB-dependent signalling pathway could be crucial for the initial cellular responses to *Orientia* via the IP challenge route. As early as 2 hpi, genes responsible for the reorganization of cell-cell interaction and cell surface were differentially expressed, possibly due to the invasion of *Orientia*. As the infection continues, genes involved in additional pathways were differentially expressed. For example, the TGFβ signalling pathway and extracellular matrix–receptor interaction pathway were activated between 4 hpi and 8 hpi, cytokine signalling, FAS signalling, different interleukin signalling, Fcγ receptor mediated phagocytosis and Fcγ receptor signalling were all significantly regulated during 12 hpi to 18 hpi. These are consistent with the idea that early gene regulation involved *Orientia* invasion and host cellular immune responses.

During the late phase, from 1 dpi and 4 dpi, pathways related to many cellular functions were affected. In addition to pathways involved in cellular immune responses and MAPK/JAK_STAT pathways, several metabolic pathways were significantly affected, such as pyruvate metabolism and TCA cycle and respiratory electron transfer, bile acid metabolism, metabolism of lipids and lipoproteins, fatty acid β-oxidation, metabolism of amino acids, and derivatives, arachidonic acid metabolism, and tryptophan metabolism. These observations suggest that as the infection progressed, host cellular responses shifted from establishment of infection to affecting host metabolic pathways to potentially accommodate the need of *Orientia* growth. Specifically, we observed that *Ido2* was one of the up-regulated genes at 2 dpi and its gene product is known to catabolize tryptophan to kynurenine. This is of interest because indoleamine 2,3-dioxygenase (*Ido1*), another gene product involved in catabolizing of tryptophan, is up-regulated in scrub typhus patients [54]. Although *Ido1* was not observed in this study, it is possible that a different route of infection for the two hosts (i.e., human and mouse) may explain the difference in observation of either *Ido1* or *Ido2* up-regulation in *Orientia* infected hosts. Many genes participating in fatty acid β-oxidation were also up-regulated, including Hmgcs2, that is involved in the ketogenesis pathway leading up to the generation of 3-hydroxybutyrate. Interestingly enough, the accumulation of 3-hydroxybutyrate has been observed in two metabolic studies in *Orientia* infected mouse models [55, 56]. These results demonstrated a significant perturbation of energy production.

Antigen presenting and processing, and Class I MHC mediated antigen presenting and processingwere significantly regulated at 7 dpi. These pathways are particularly interesting since antibody responses were also detectable after 7 dpi. In addition, genes such as *Il15ra* (1.5 folds), *Il12rb1* (4.1 folds), *Il18* (2.7 folds) were significantly up-regulated only at 7 dpi. Since these cytokines are all IFNγ-induced cytokines, it is not surprising that these cytokines are released into serum samples in high concentrations in scrub typhus patients [57]. Furthermore, the transcriptomic study of monocytes infected by *Orientia* had shown many genes up-regulated at 8 hpi and 1 dpi, including, *Il-1b*, *Cxcl10*, *Oas1*, and *Mx1* [58]. These genes were also observed significantly up-regulated by 3.6, 16.0, 7.5 and 16.0 folds at 7 dpi, respectively. It is not clear whether any of these genes were specifically responsible for the overall outcome. Nevertheless, the combination of these genes, when they are all significantly up- or down-regulated simultaneously, is likely to be the major contributing factor for the final outcome of *Orientia* infection.

It is noted that IP is not a natural route for infection nor is the amount of live *Orientia* used be comparable with the chigger-fed mouse model [59]. Regardless, there are similarities between the two models that could still provide information regarding cellular responses to *Orientia* infection. For example, in both cases, the time for onset of symptoms is around 9 dpi, and both routes resulted in animal death within 21 dpi. On the contrary, multiple variables are different between the two models. For example, the infection by chiggers may also involve an exchange of proteins between chigger and animal host, while IP injection does not. Consequently, it is important to explore the regulatory pathways in a chigger challenge model in order to better understand the cellular regulation upon *Orientia* infection.

## Conclusions

Many pathways are dynamically regulated at different time points post *Orientia* IP infection in a mouse model. During the initial phase of infection, genes involved in pathways related to cell growth control, cytokine secretion, cell motility, extracellular matrix adhesion, cell transformation and invasion, and cell development are significantly regulated, supporting the idea that the *Orientia* is actively affecting the host in order to survive. At later time post-inoculation, genes involved in cellular immune responses are triggered to defend infection and try to eliminate the pathogen. We observed good correlation between regulation of various metabolic pathways and the change of amounts of various transcripts and proteins involved in them, suggesting that the growth of *Orientia* after initial invasion affected many different metabolic activities. Furthermore, the regulation of complement and coagulation pathways may play a role in the overall homeostasis of pathogens and host immune responses.

## Methods

### Mouse model

**Fig. 8:**
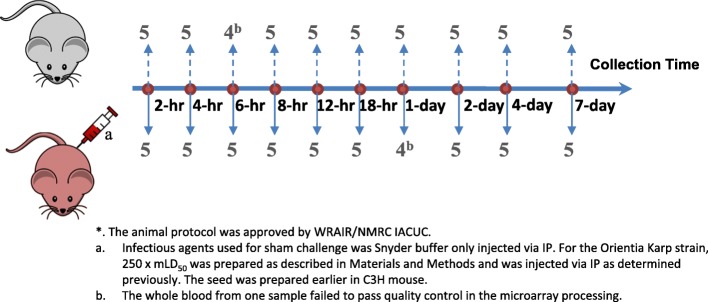
Study Design. A total of 5 mice per group received Snyder buffer or *Orientia* Karp strain at indicated dosage via IP. Blood was taken at 10 different time points after IP inoculation. Blood were taken from each mouse as described in Material and Methods

The procedures for animal study were approved by the Walter Reed Army Institute of Research (WRAIR) Naval Medical Research Center (NMRC) Institutional Animal Care and Use Committee (IACUC). The facility’s animal care and use program is accredited by Association for Assessment and Accreditation of Laboratory Animal Care International (AAALAC). Female eight-week old C3HeB/FeJ (C3H) inbred mice were purchased from Jackson Laboratories (Bar Harbor, Maine). Mice were divided into two groups and housed in an ABSL3 laboratory in cages (5 mice per cage) in accordance with the most current SOPs established by Veterinary Service Program (VSP) of NMRC. Feeding and water were provided *ad libidum*. One group of mice was inoculated with Snyder’s buffer (sham) to serve as naïve controls and those in the other group (infected) were infected via IP route [28] with 250 mLD_50_ of liver/spleen homogenate from mice infected by the Karp strain *O. tsutsugamushi* from New Guinea as described previously [60]. We used Snyder’s buffer as a negative control instead of normal non-*Orienita* infected mouse liver-spleen homogenate based on results obtained from previously performed experiments (data not shown). The results from these experiments showed that sera from normal liver-spleen homogenate inoculated mice exhibited similar amounts of Th1 (TNF-α, IFN-γ, IL-2, and IL-12) and Th2 (IL-4, IL-5, and IL-10) cytokines as those from sera collected from sham inoculated mice. Therefore, the results suggest that only limited if any immune responses are activated by liver-spleen homogenate from uninfected mice. After inoculation, mice were observed twice daily for up to 12 days. Clinical signs, such as ruffled fur, mobility and morbidity were recorded. Each group of mice was divided into 5 mice per time point for a total of 10 different time points (2-h (hr), 4-h, 6-h, 8-h, 12-h, 18-h, 24-h, 2-day, 4-day and 7-day, Fig. 8). At the indicated times, mice from each group were euthanized by CO_2_, blood was drawn (detail described below) followed by collecting lung, liver, and spleen. Five additional mice were infected with the same infectious dose and monitored daily for up to 21 days or until they succumb to infection to ensure that 250 mLD_50_ is a lethal.

### Blood collection, RNA extraction and data acquisition at indicated times

Before euthanasia whole blood from each mouse was collected by cardiac puncture. A total of 0.6 mL blood was immediately transferred to PAXgene™ blood RNA Tubes (Qiagen, Valencia, CA, USA). The samples were mixed and then flash frozen in liquid nitrogen until use. Additional blood was centrifuged to obtain serum for serology. RNA extraction was performed on a Qiacube™ (Qiagen, Valencia, CA, USA) and the quantity of extracted RNA was measured by NanoDrop™ 2000 spectrometer (Thermo Fisher Scientific, Waltham, MA, USA) as per manufacturer’s guideline. Agilent® SurePrint G3 Mouse GE 8x60K Microarray slides with associated Feature Extraction software v11.0.1.1 (Agilent Technologies, Santa Clara, CA, USA) were used following recommended protocol. Each SurePrint G3 Mouse GE 8x60K Microarray slide contains 27,122 RefSeq (Entrez) gene count and 4578 lncRNA transcripts. All genes have multiple probes.

### Recombinant protein antigen based ELISA for antibody detection

ELISA was performed as previously described [61]. The cutoff was determined by using the mean of the sham group plus 1.745 times the standard deviation for the 95% confidence interval [62].

### Detection of soluble cell adhesion molecules (sCAMs)

Serum samples from all time points were assessed for sL-selectin, sICAM-1 and sVCAM-1 by using ELISA kits (R & D Systems, Minneapolis, MN, USA) following the manufacturer’s instructions. Samples were assayed in duplicate. The optical density at 465 nm (Vmax/Kinetic Microplate Reader, Molecular Devices, San Jose, CA, USA) was measured. The quantity of each molecule was calculated based on a standard curve established with known amount of the said molecule. The average quantity of each molecule in mouse sera from sham inoculated mice and from *Orientia* inoculated mice was compared by t-test at each time point.

### Quantitation of O. tsutsugamushi

The quantity of *O. tsutsugamushi* in liver, lung, and spleen after inoculation was determined by a qPCR assay targeting the *Orientia*-specific 47 kDa gene [63]. Mouse-specific primers for the single copy gene mouse complement factor D (cfd) were used to quantitate the amount of mouse gene equivalents in the extracted DNA as described by Sunyakumthorn et al. [29].

### RT-qPCR processing

A real-time quantitative PCR (RT-qPCR) analysis was carried out using the RT^2^ Profiler PCR Array System (SA Biosciences Corp, Frederick, MD) focusing on the blood clotting cascade and classical complement pathway according to the manufacturer’s protocol. Five hundred ng of total RNA was used to produce cDNA using the RT^2^ First Strand Kit (Qiagen, Germantown, MD, USA), followed by qPCR assays using the RT^2^ SYBR Green/Rox Mastermix Kit (Qiagen, Germantown, MD, USA) in an Applied Biosystems 7900HT (Applied Biosystems, Foster City, CA, USA) under the recommended conditions. The RT-qPCR results were analyzed with SDS 2.3 software (Applied Biosystems, Foster City, CA, USA).

### Data pre-processing to identify differentially expressed genes

For those samples that failed in RNA extraction or had a low RIN (RIN < 6.5), they were removed from microarray experiment. All data from microarray experiment was preprocessed and quality control was performed using within-chip Lowess and between-chip quantile normalization. Two outliers were identified by Principal Component Analysis and were removed from downstream analysis as they could bias the analysis. Batch correction of extraction time was done using Bioconductor limma 3.7 package [64] in R 3.2 [65]*.* Differentially expressed genes (DEGs) were identified using Wilcoxon rank-sum test.

### Clustering and enrichment analysis

The clustering and pathway analysis were performed using Functional Heatmap (https://bioinfo-abcc.ncifcrf.gov/Heatmap/index.php) and custom R code. Functional Heatmap first transfers gene expression levels into average fold change curves, and then transfers the changes above 0.5 as symbol “+”, below − 0.5 as symbol “-“, and the rest as “0”. Thus we generalized the curves into strings. Finally, the strings with similar appearance were grouped into patterns and the genes of selected patterns were sent to pathway enrichment analysis using the hypergeometric test. The pathway view showed the temporal pathway expression for known pathways collected from five pathway databases KEGG version 80 [66], Wiki pathway [67], Biocarta [68], Reactome [69], and GSEA [70]. The dark green, green, red, and dark red stand for the percentage of the genes with log2 fold change of (−∞, − 1), (− 1,-0.5), (0.5, 1), and (1, +∞), respectively. Pathways with 95% common components were merged.

## Supplementary information

**Additional file 1: Table S1.** Enriched Pathways (K: KEGG, R: Reactome, B: Biocarta, W: Wikipathways, H: Hallmark genes; ST: Signal Transduction; | Nearly identical pathways).

## Data Availability

The datasets used and analyzed during the current study are available from the corresponding author upon reasonable request. The results from analysis of the datasets are presented in this published article as tables, figures and supplementary information files.

## Abbreviations

AAALAC: Association for Assessment and Accreditation of Laboratory Animal Care International
ABSL3: Animal biosafety level 3
C3H: C3HeB/FeJ
cfd: complement factor D
DEPs: Differential expressed probes
dpi: days post inoculation
GSEA: Gene set enrichment analysis
h: hour
IACUC: Institutional Animal Care and Use Committee
ID: Intradermal
IP: Intraperitoneal
IV: Intravenous
KEGG: Kyoto Encyclopedia of Genes and Genomes
mLD50: lethal dosage causing 50% death in a mouse
NMRC: Naval Medical Research Center
hpi: hours post inoculation
sCAM: soluble cell adhesion molecules
sICAM-1: soluble intercellular adhesion molecule-1
SOPs: Standard operation procedures
sVCAM-1: soluble vascular cell adhesion molecule-1
VSP: Veterinary services program
