# Supplementary material for: Temporal analysis of mRNA expression profiles in Orientia infected C3HeB/FeJ mouse
Source: BMC Microbiol. 2020 Jan 6;20:3. doi: 10.1186/s12866-019-1684-3 (PMC6945539; doi:10.1186/s12866-019-1684-3)
Supplement: Supplementary file 1 — Additional file 1: Table S1. Enriched Pathways (K: KEGG, R: Reactome, B: Biocarta, W: Wikipathways, H: Hallmark genes; ST: Signal Transduction; | Nearly identical pathways). [file 12866_2019_1684_MOESM1_ESM.docx]

**Table S1: Enriched Pathways (K: KEGG, R: Reactome, B: Biocarta, W: Wikipathways, H: Hallmark genes; ST: Signal Transduction; | Nearly identical pathways)**

| Time | Enriched Pathways |
| --- | --- |
| Hr2 | W_Integrated_Cancer\|W_Integrated_Breast_Cance...  H_TNFA_SIGNALING_VIA_NFKB  H_HYPOXIA  R_G_alpha_(12/13)_sign._events  B_RAC1  H_MYC_TARGETS_V1  R_Inact._of_Cdc42_and_Rac  K_PPAR_sign.  B_P35ALZHEIMERS  K_Epithelial_cell_sign._in_Helicobacter_pylor...  B_ERK\|B_CDK5  W_Homologous_recombination\|K_Homologous_recom...  W_Adipogenesis  ST_ERK1_ERK2_MAPK  W_TOR_sign.\|R_Reg._of_Rheb_GTPase_activity_by...  W_Mitochondrial\|R_Organelle_biogenesis_and_ma...  K_Acute_myeloid_leukemia  B_TALL1  B_NUCLEARRS\|W_Nuclear_recep._in_lipid_metab._...  H_ADIPOGENESIS  H_KRAS_SIGNALING_UP |
| Hr4 | W_Adipocyte_TarBase\|W_Epithelium_TarBase\|W_Ly...  R_Sign._by_Hippo  R_DNA_Repair\|R_Homologous_recombination_repai...  R_Acetylcholine_Binding_And_Downstream_Events...  B_CARDIACEGF  W_Leukocyte_TarBase  R_Nucleotide_Excision_Repair\|R_Global_Genomic...  H_TGF_BETA_SIGNALING  B_CHEMICAL  R_Nephrin_interact.s  H_E2F_TARGETS\|SA_G2_AND_M_PHASES  H_DNA_REPAIR  K_Viral_carcinogenesis  K_Glycosylphosphatidylinositol(GPI)-anchor_bi...  H_UV_RESPONSE_UP  R_Interaction_between_L1_and_Ankyrins  K_s_in_cancer\|K_Endometrial_cancer\|K_Thyroid_...  R_Regulatory_RNAs\|R_MicroRNA_(miRNA)_biogenes...  K_Neuroactive_ligand-receptor_interact.\|R_His... |
| Hr6 | W_Nicotine_Activity_on_Dopaminergic_Neurons\|K...  W_Leukocyte_TarBase  W_Statin_  R_G_alpha_(12/13)_sign._events  SA_PROGRAMMED_CELL_DEATH\|W_Apoptosis_Modulati...  R_Act._of_Kainate_Recep._upon_glutamate_bindi...  K_Protein_digestion_and_absorption  K_ECM-receptor_interact.\|R_Laminin_interact.s...  H_HEDGEHOG_SIGNALING  B_AGR  B_GABA\|K_GABAergic_synapse  R_L1CAM_interact.s\|R_CHL1_interact.s  R_Non-integrin_membrane-ECM_interact.s\|R_Synd...  R_Transcriptional_activity_of_SMAD2/SMAD3:SMA...  R_MHC_class_II_antigen_presentation  K_Transcriptional_misreg._in_cancer  K_Morphine_addiction\|R_GABA_receptor_act.\|R_G... |
| Hr8 | R_Neuronal_System\|R_Transmission_across_Elect...  R_Bicarbonate_transporters  R_SLC-mediated_transmembrane_transport\|R_Cati...  R_Neurotransmitter_Receptor_Binding_And_Downs...  H_KRAS_SIGNALING_DN  R_CRMPs_in_Sema3A_sign.  R_Potassium_Channels\|R_Tandem_pore_domain_pot...  R_Sign._by_NODAL\|R_Reg._of_sign._by_NODAL  K_Long-term_potentiation\|R_Act._of_NMDA_recep...  K_TGF-beta_sign.\|R_Sign._by_Activin |
| Hr12 | H_APOPTOSIS  ST_GA13  K_Vascular_smooth_muscle_contraction  B_STEM  R_Acetylcholine_Binding_And_Downstream_Events...  R_Proc._of_Capped_Intron-Containing_Pre-mRNA\|...  W_Nucleotide_Metab.  SIG_CHEMOTAXIS\|SIG_REGULATION_OF_THE_ACTIN_CY...  ST_GA12  B_AT1R\|B_PYK2\|K_GnRH_sign.  R_Cytokine_Sign._in_Immune_system\|R_IL-1_proc...  ST_FAS_SIGNALING  R_Antiviral_mechanism_by_IFN-stimulated_genes...  K_N-Glycan_biosyn.\|R_Asparagine_N-linked_glyc...  R_Semaphorin_interact.s\|R_Sema4D_in_semaphori...  W_Osteopontin_Sign.\|W_TWEAK_Sign.\|K_TNF_sign....  B_DEATH\|W_Apoptosis\|K_Apoptosis_Cell\|R_Apopto...  R_Chromatin_organization\|R_Chromatin_modifyin...  SA_PROGRAMMED_CELL_DEATH\|W_Apoptosis_Modulati... |
| Hr18 | W_IL-5_sign.  B_VDR\|K_Thyroid_hormone_sign.  R_DAP12_sign.\|R_DAG_and_IP3_sign.\|R_CaM\|R_Cal...  B_RARRXR  W_Nifedipine_Activity\|W_EGF/EGFR_Sign._  B_NFAT  B_IL2\|B_IL2RB\|W_IL-2_Sign.  W_IL-7_sign.\|W_IL-9_sign.  R_Sign._by_NGF\|R_p75_NTR_receptor-mediated_si...  H_MTORC1_SIGNALING  R_Developmental_Biology\|R_Axon_guidance\|R_Neu...  R_FCERI_mediated_Ca+2_mobilization  K_Acute_myeloid_leukemia  R_Fcgamma_receptor_(FCGR)_dep._phagocytosis\|R...  R_NGF_sign._via_TRKA_from_the_plasma_membrane...  W_Insulin_Sign.\|K_mTOR_sign.\|R_RSK_act.  R_Transcriptional_reg._of_white_adipocyte_dif...  W_Physiological_and_Pathological_Hypertrophy_...  R_DAP12_interact.s\|R_Downstream_sign._of_acti...  B_IGF1R\|K_Insulin_sign.  K_Long-term_potentiation\|R_Act._of_NMDA_recep...  B_SHH  B_CREB  W_Kit_receptor_sign._  B_EGF\|B_IGF1\|B_GLEEVEC\|B_INSULIN\|B_NGF\|B_PDGF...  H_PI3K_AKT_MTOR_SIGNALING  W_Mitochondrial\|R_Organelle_biogenesis_and_ma...  K_Gap_junction  B_MAL  R_Reg._of_lipid_metab._by_Peroxisome_prolifer...  R_Fc_epsilon_receptor_(FCERI)_sign.\|R_FCERI_m...  K_Fc_gamma_R-mediated_phagocytosis  R_Sign._by_FGFR\|R_Negative_reg._of_FGFR_sign....  B_BIOPEPTIDES  R_NCAM_sign._for_neurite_out-growth\|R_RAF/MAP...  B_IGF1MTOR  B_RHO  W_IL-6_sign.\|R_IL-6_sign.  B_PPARA\|B_CARM1  K_Biosyn._of_unsaturated_fatty_acids  B_EGFR_SMRTE  R_L1CAM_interact.s\|R_CHL1_interact.s  B_SALMONELLA\|B_CDC42RAC\|B_ACTINY\|K_Reg._of_ac...  B_BCR\|B_CALCINEURIN\|B_FCER1\|B_GPCR\|B_TCR  B_ERK5\|W_B_Cell_Receptor_Sign.  B_CK1\|R_DARPP-32_events  R_Sign._by_Insulin_receptor\|R_IRS_act.\|R_Sign...  B_PTDINS  R_Sign._to_ERKs\|R_Sign._to_RAS\|R_p38MAPK_even...  B_NOS1  R_Growth_hormone_receptor_sign.  R_Sign._by_EGFR\|R_EGFR_downreg.  W_TSH_sign._  W_Leptin_sign.\|R_Sign._by_Leptin  R_Sign._by_SCF-KIT\|R_Reg._of_KIT_sign.  K_s_in_cancer\|K_Endometrial_cancer\|K_Thyroid_...  K_Neurotrophin_sign.\|R_Sign._to_p38_via_RIT_a...  B_FMLP  B_MET\|W_Sign._of_Hepatocyte_Growth_Factor_Rec...  K_B_cell_receptor_sign.  K_T_cell_receptor_sign.\|R_Act._of_Rac  R_Recycling_of_L1  R_Cell_Cycle,_Mitotic\|R_Nuclear_Envelope_Brea...  B_GH  W_Calcium_Reg._in_the_Cardiac_Cell\|K_Circadia...  W_ErbB_sign._\|K_ErbB_sign.  B_RANKL\|K_Osteoclast_diff.  B_VIP  B_ECM\|W_Integrin-mediated_cell_adhesion\|W_Foc...  R_IRS-related_events\|R_IRS-mediated_sign.\|R_P...  K_Vasopressin-regulated_water_reabsorption  W_EPO_Receptor_Sign.\|K_Prolactin_sign.  R_Sign._by_ILs\|R_IL-2_sign.\|R_IL_receptor_SHC...  H_UV_RESPONSE_UP  W_Integrated_Cancer\|W_Integrated_Breast_Cance...  B_TRKA\|SA_TRKA_RECEPTOR\|K_Proteoglycans_in_ca...  B_ERK\|B_CDK5  R_Oncogene_Induced_Senescence  B_PTEN  B_BARR_MAPK\|B_BARRESTIN_SRC\|B_BARRESTIN  B_SPRY  K_Vascular_smooth_muscle_contraction  W_angiogenesis_overview  R_Cell_Cycle\|R_Reg._of_mitotic_cell_cycle\|R_P...  R_Factors_involved_in_megakaryocyte_developme...  K_Renal_cell_carcinoma  K_Ribosome_biogenesis_in_eukaryotes  R_Fatty_acid,_triacylglycerol,_and_ketone_bod...  K_Base_excision_repair\|R_Base_Excision_Repair...  B_LEPTIN\|W_AMPK_sign.  W_Osteopontin_Sign.\|W_TWEAK_Sign.\|K_TNF_sign....  B_MAPK\|R_Act._of_the_AP-1_family_of_transcrip...  R_Metab._of_nitric_oxide\|R_Tetrahydrobiopteri...  B_IL4\|W_IL-4_sign.  B_CDMAC\|K_Hepatitis_B  R_Establishment_of_Sister_Chromatid_Cohesion\|...  K_Rap1signaling\|R_Sign._by_VEGF  B_NDKDYNAMIN  H_HEDGEHOG_SIGNALING  K_Spliceosome  R_Prolactin_receptor_sign.\|R_Sign._by_ERBB4  W_Alzheimers_Disease\|K_Alzheimer's_disease  R_Circadian_Clock  B_CHEMICAL  B_HER2  R_Signal_transduction_by_L1  R_Reg._of_PLK1_Activity_at_G2/M_Transition\|R_...  R_Mitotic_Prometaphase\|R_Condensation_of_Prom...  K_Chronic_myeloid_leukemia  R_Innate_Immune_System\|R_TRAF3-dep._IRF_act.\|...  R_Aquaporin-mediated_transport\|R_Passive_tran...  R_M_Phase\|R_Clearance_of_Nuclear_Envelope_Mem...  K_Bladder_cancer  H_MYC_TARGETS_V1  B_CCR3  W_G13_Sign._  B_RACCYCD\|K_Prostate_cancer  W_G_Protein_Sign.\|R_Cam-PDE_1_act.  ST_INTERFERON_GAMMA  K_Bacterial_invasion_of_epithelial_cells  B_CXCR4  K_Dorso-ventral_axis_formation  H_MYC_TARGETS_V2  R_Sign._by_the_B_Cell_Receptor_(BCR)\|R_Downst...  B_CFTR\|B_MPR\|B_PLCE |
| Day1 | H_PROTEIN_SECRETION  H_EPITHELIAL_MESENCHYMAL_TRANSITION  R_Transcriptional_activity_of_SMAD2/SMAD3:SMA...  R_trans-Golgi_Network_Vesicle_Budding  R_Amino_acid_syn._and_interconversion_(transa...  ST_JAK_STAT\|ST_STAT3  R_Sign._by_TGF-beta_Receptor_Complex_in_Cance...  W_SREBP_sign.\|K_Protein_proc._in_endoplasmic_...  W_MAPK_sign._\|W_MAPK_Cascade\|K_MAPK_sign.\|R_A...  K_Prion_diseases  K_TGF-beta_sign.\|R_Sign._by_Activin  W_Adipocyte_TarBase\|W_Epithelium_TarBase\|W_Ly...  K_Inositol_phosphate_metab.\|K_Phosphatidylino...  K_Mineral_absorption  W_Type_II_diabetes_mellitus\|W_Nicotine_Activi...  W_TarBase  W_SIDS_Susceptibility  K_Melanoma  B_SALMONELLA\|B_CDC42RAC\|B_ACTINY\|K_Reg._of_ac...  H_UV_RESPONSE_DN  K_Dorso-ventral_axis_formation  R_Nef-mediates_down_modulation_of_cell_surfac...  B_PS1  K_ABC_transporters\|R_ABC-family_proteins_medi...  K_SNARE_interact.s_in_vesicular_transport  R_Sign._by_Insulin_receptor\|R_IRS_act.\|R_Sign...  K_Fatty_acid_biosyn.  W_Triacylglyceride_Synthesis\|K_Glycerolipid_m...  B_CDMAC\|K_Hepatitis_B  R_Sign._by_EGFR\|R_EGFR_downreg.  B_PTC1\|R_G2/M_Transition\|R_Cyclin_A/B1_associ...  R_Prolactin_receptor_sign.\|R_Sign._by_ERBB4  K_N-Glycan_biosyn.\|R_Asparagine_N-linked_glyc...  R_Sign._by_Rho_GTPases\|R_Rho_GTPase_cycle  W_ID_sign._  W_Serotonin_Receptor_2_and_ELK-SRF/GATA4_sign...  H_ESTROGEN_RESPONSE_EARLY  R_Phospholipid_metab.\|R_PI_Metab.  W_Arylamine_metab.\|K_Chemical_carcinogenesis  W_IL-5_sign.  B_RACCYCD\|K_Prostate_cancer |
| Day2 | K_Propanoate_metab.\|R_Metab.\|R_Mitochondrial_...  H_FATTY_ACID_METABOLISM\|W_Fatty_Acid_Beta_Oxi...  H_XENOBIOTIC_METABOLISM  H_BILE_ACID_METABOLISM\|K_Primary_bile_acid_bi...  R_Biological_oxidations\|R_Amino_Acid_conjugat...  R_Metab._of_lipids_and_lipoproteins\|R_Lipopro...  W_Tryptophan_metab.\|K_Tryptophan_metab.\|R_Try...  W_cytochrome_P450\|R_Vitamins  R_Bile_acid_and_bile_salt_metab.\|R_Recycling_...  W_Catalytic_cycle_of_mammalian_FMOs\|W_meta_bi...  W_Fatty_Acid_Omega_Oxidation\|R_Phase_1_-_Func...  R_Metab._of_amino_acids_and_derivatives\|R_Car...  H_PEROXISOME  K_Peroxisome\|R_Peroxisomal_lipid_metab.  K_Valine_leucine_and_isoleucine_degrad.\|R_Bra...  W_Codeine_and_morphine_metab.\|K_Bile_secretio...  W_Glucocorticoid_&_Mineralcorticoid_Metab.\|K_...  K_Retinol_metab.\|R_Heme_degrad.  W_Tamoxifen_metab.  W_Aflatoxin_B1_metab.\|K_Metab._of_xenobiotics...  R_Xenobiotics\|R_CYP2E1_reactions  W_Arylamine_metab.\|K_Chemical_carcinogenesis  H_ADIPOGENESIS  K_PPAR_sign.  W_Methylation\|R_Phase_II_conjugation\|R_Methyl...  W_Synthesis_and_Degrad._of_Ketone_Bodies\|K_Sy...  W_Glucuronidation\|K_Pentose_and_glucuronate_i...  W_Urea_cycle_and_metab._of_amino_groups\|K_Arg...  W_Steroid_Biosyn.  W_Nicotine_metab.\|K_Drug_metab._-_cytochrome_...  R_Fatty_acid,_triacylglycerol,_and_ketone_bod...  B_NUCLEARRS\|W_Nuclear_recep._in_lipid_metab._...  W_Selenium_\|W_Vitamin_B12_Metab.\|W_Folate_Met...  R_Reg._of_cholesterol_biosyn._by_SREBP_(SREBF...  H_OXIDATIVE_PHOSPHORYLATION\|B_KREB\|W_TCA_Cycl...  H_MTORC1_SIGNALING  H_GLYCOLYSIS  K_Glyoxylate_and_dicarboxylate_metab.  H_CHOLESTEROL_HOMEOSTASIS\|W_Cholesterol_Biosy...  W_Statin_  R_Endogenous_sterols  K_beta-Alanine_metab.  R_Amino_acid_syn._and_interconversion_(transa...  W_Fluoropyrimidine_Activity  K_Biosyn._of_unsaturated_fatty_acids  W_Electron_Transport_Chain\|W_Oxidative_phosph...  W_Fatty_Acid_Biosyn.  K_Fatty_acid_elongation  R_Metab._of_vitamins_and_cofactors\|R_Molybden...  R_Biotin_transport_and_metab.\|R_Defects_in_vi...  K_Pyruvate_metab.  R_Arachidonic_acid_metab.\|R_Fatty_acids\|R_Eic...  R_Sulfur_amino_acid_metab.\|R_Degrad._of_cyste...  K_Drug_metab._-_other_enzymes  H_COAGULATION\|B_FIBRINOLYSIS\|W_Blood_Clotting...  R_The_citric_acid_(TCA)_cycle_and_respiratory...  W_Irinotecan_  R_Reg._of_lipid_metab._by_Peroxisome_prolifer...  K_Maturity_onset_diabetes_of_the_young\|R_Reg....  R_Lipid_digestion,_mobilization,_and_transpor...  R_Transport_of_vitamins,_nucleosides,_and_rel...  W_Alanine_and_aspartate_metab.\|K_Alanine_aspa...  B_CARDIACEGF  W_Triacylglyceride_Synthesis\|K_Glycerolipid_m...  K_Steroid_biosyn.  W_Arachidonate_Epoxygenase_/_Epoxide_Hydrolas...  K_Arachidonic_acid_metab.\|K_Linoleic_acid_met...  R_Diseases_associated_with_visual_transductio...  H_COMPLEMENT  R_Glucose_metab.\|R_Glycogen_syn.  K_Lysine_degrad.\|R_Lysine_catabolism  R_Metab._of_folate_and_pterines  K_Histidine_metab.\|R_Histidine_catabolism  R_PTM:_gamma_carboxylation,_hypusine_formatio...  R_Transport_of_organic_anions  R_Metab._of_steroid_hormones_and_vitamin_D\|R_...  W_Glutathione_metab.\|K_Glutathione_metab.\|R_G...  W_Nuclear_Recep.\|R_Nuclear_Receptor_transcrip...  R_Reg._of_beta-cell_development\|R_Reg._of_gen...  R_Formation_of_Fibrin_Clot_(Clotting_Cascade)...  B_ACE2  W_Osteoblast_Sign.  K_Bladder_cancer  R_Integrin_cell_surface_interact.s  W_Selenium_Metab._and_Selenoproteins  R_Amine-derived_hormones  W_ACE_Inhibitor_  W_Estrogen_metab.  K_Selenocompound_metab.  K_Renin-angiotensin_system  R_SLC-mediated_transmembrane_transport\|R_Cati...  R_Retinoid_metab._and_transport  W_Heme_Biosyn.\|K_Porphyrin_and_chlorophyll_me...  K_Adherens_junction  B_MTA3  K_Cardiac_muscle_contraction\|K_Adrenergic_sig...  B_ETC\|K_Non-alcoholic_fatty_liver_disease_(NA... |
| Day4 | K_Proximal_tubule_bicarbonate_reclamation  R_Biological_oxidations\|R_Amino_Acid_conjugat...  W_Catalytic_cycle_of_mammalian_FMOs\|W_meta_bi...  H_PEROXISOME  H_G2M_CHECKPOINT  H_KRAS_SIGNALING_DN  B_MITOCHONDRIA\|R_Intrinsic_Pathway_for_Apopto...  H_ALLOGRAFT_REJECTION\|B_ASBCELL\|B_TH1TH2  K_Propanoate_metab.\|R_Metab.\|R_Mitochondrial_...  W_Adipogenesis |
| Day7 | R_Cell_Cycle,_Mitotic\|R_Nuclear_Envelope_Brea...  R_Cell_Cycle\|R_Reg._of_mitotic_cell_cycle\|R_P...  H_E2F_TARGETS\|SA_G2_AND_M_PHASES  R_M_Phase\|R_Clearance_of_Nuclear_Envelope_Mem...  H_G2M_CHECKPOINT  H_MYC_TARGETS_V1  H_INTERFERON_ALPHA_RESPONSE\|H_INTERFERON_GAMM...  W_DNA_Replication\|R_Act._of_the_pre-replicati...  R_G1/S_Transition\|R_G1/S-Specific_Transcripti...  R_Mitotic_G1-G1/S_phases\|R_Reg._of_DNA_replic...  R_S_Phase\|R_Unwinding_of_DNA  B_MCM\|R_Cell_Cycle_Checkpoints\|R_Act._of_ATR_...  R_Mitotic_Spindle_Checkpoint\|R_Act._of_APC/C_...  B_DEATH\|W_Apoptosis\|K_Apoptosis_Cell\|R_Apopto...  R_Immune_System\|R_Complement_cascade\|R_Altern...  R_Mitotic_Prometaphase\|R_Condensation_of_Prom...  B_PROTEASOME\|W_Proteasome_Degrad.\|K_Proteasom...  R_Antigen_proc.-Cross_presentation\|R_Endosoma...  R_Establishment_of_Sister_Chromatid_Cohesion\|...  R_Reg._of_mRNA_stability_by_proteins_that_bin...  R_HIV_Infection\|R_Nef_and_signal_transduction...  R_Reg._of_Apoptosis\|R_Role_of_DCC_in_regulati...  H_MTORC1_SIGNALING  K_Spliceosome  B_CD40\|B_TNFR2\|K_Epstein-Barr_virus_infection...  R_TCF_dep._sign._in_response_to_WNT\|R_WNT_med...  R_Class_I_MHC_mediated_antigen_proc._&_pr...  R_Sign._by_Wnt\|R_formation_of_the_beta-cateni...  R_Adaptive_Immune_System\|R_Role_of_LAT2/NTAL/...  R_Cytokine_Sign._in_Immune_system\|R_IL-1_proc...  R_Chromosome_Maintenance\|R_Extension_of_Telom...  H_MITOTIC_SPINDLE  B_G1\|B_CELLCYCLE\|SA_REG_CASCADE_OF_CYCLIN_EXP...  R_beta-catenin_indep._WNT_sign.\|R_PCP/CE\|R_WN...  B_G2\|B_RB\|K_Cell_cycle_Cell\|R_Cyclin_B2_media...  R_Disease\|R_Neurotoxicity_of_clostridium_toxi...  K_Oocyte_meiosis_Cell  R_Reg._of_PLK1_Activity_at_G2/M_Transition\|R_...  R_Cellular_responses_to_stress\|R_Cellular_res...  B_RELA\|K_Herpes_simplex_infection  B_HCMV\|K_Influenza_A  K_DNA_replication\|K_Nucleotide_excision_repai...  B_PTC1\|R_G2/M_Transition\|R_Cyclin_A/B1_associ...  R_Mitotic_G2-G2/M_phases\|R_Polo-like_kinase_m...  R_Interferon_Sign.\|R_Interferon_gamma_sign.\|R...  H_TNFA_SIGNALING_VIA_NFKB  H_ALLOGRAFT_REJECTION\|B_ASBCELL\|B_TH1TH2  K_Propanoate_metab.\|R_Metab.\|R_Mitochondrial_...  R_Nucleosome_assembly\|R_Deposition_of_new_CEN...  R_Sign._by_the_B_Cell_Receptor_(BCR)\|R_Downst...  H_IL6_JAK_STAT3_SIGNALING  R_Metab._of_nucleotides\|R_Phosphate_bond_hydr...  R_Proc._of_Capped_Intron-Containing_Pre-mRNA\|...  W_Leukocyte_TarBase  K_Pathogenic_Escherichia_coli_infection  K_Legionellosis  R_Metab._of_amino_acids_and_derivatives\|R_Car...  B_RNA\|K_Measles  W_Alzheimers_Disease\|K_Alzheimer's_disease  R_RNA_Polymerase_I_Transcription\|R_Epigenetic...  R_Protein_folding\|R_Post-chaperonin_tubulin_f...  K_Malaria  B_ETC\|K_Non-alcoholic_fatty_liver_disease_(NA...  W_mRNA_proc.  B_IL7\|K_HTLV-I_infection  R_Cellular_Senescence\|R_DNA_Damage/Telomere_S...  K_Viral_carcinogenesis  B_TOLL\|W_Reg._of_toll-like_receptor_sign.\|W_N...  W_miRNA_reg._of_DNA_Damage_Response\|W_miRNAs_...  H_DNA_REPAIR  H_INFLAMMATORY_RESPONSE  H_GLYCOLYSIS  R_Senescence-Associated_Secretory_Phenotype_(...  K_Pyrimidine_metab._Nucleotide_metab.\|R_Pyrim...  W_SREBP_sign.\|K_Protein_proc._in_endoplasmic_...  K_RNA_transport\|R_Hexose_transport\|R_Glucose_...  K_Progesterone-mediated_oocyte_maturation  W_NOD  K_NOD-like_receptor_sign.  H_UNFOLDED_PROTEIN_RESPONSE  H_APOPTOSIS  W_Osteopontin_Sign.\|W_TWEAK_Sign.\|K_TNF_sign....  B_NKCELLS\|K_Natural_killer_cell_mediated_cyto...  R_Gene_Expression\|R_Post-transcriptional_sile...  K_Chagas_disease_(American_trypanosomiasis)  R_RNA_Polymerase_II_Transcription\|R_Proc._of_...  SIG_PIP3_SIGNALING_IN_CARDIAC_MYOCTES\|SIG_INS...  W_TFs_Regulate_miRNAs_related_to_cardiac_hype...  K_Type_II_diabetes_mellitus  R_Platelet_act.,_sign._and_aggregation\|R_GP1b...  B_MITOCHONDRIA\|R_Intrinsic_Pathway_for_Apopto...  R_Antiviral_mechanism_by_IFN-stimulated_genes...  W_Type_II_interferon_sign._(IFNG)  R_Unfolded_Protein_Response_(UPR)\|R_ATF6-alph...  K_Phagosome\|K_Vibrio_cholerae_infection\|R_Iro...  ST_TUMOR_NECROSIS_FACTOR\|W_TNF_alpha_Sign._  R_Sign._by_ILs\|R_IL-2_sign.\|R_IL_receptor_SHC...  R_TAK1_activates_NFkB_by_phosphor._and_act._o...  R_Nucleotide-binding_domain,_leucine_rich_rep...  B_RANMS  H_ADIPOGENESIS  ST_TYPE_I_INTERFERON\|K_Hepatitis_C  K_Pertussis  R_Purine_metab.  B_EIF2  R_MASTL_Facilitates_Mitotic_Progression  W_Adipocyte_TarBase\|W_Epithelium_TarBase\|W_Ly...  K_Gap_junction  H_OXIDATIVE_PHOSPHORYLATION\|B_KREB\|W_TCA_Cycl...  R_Cellular_response_to_heat_stress\|R_HSF1-dayep...  B_CLASSIC\|B_COMP\|B_LECTIN\|W_Complement_Act.\|K...  W_Apoptosis_Modulation_by_HSP70  B_IL2\|B_IL2RB\|W_IL-2_Sign.  R_Advanced_glycosylation_endproduct_receptor_...  K_Purine_metab._Nucleotide_metab.\|K_RNA_polym...  K_Chemokine_sign.\|R_Reg._of_insulin_secretion...  R_Synthesis_and_interconversion_of_nucleotide...  R_Hemostasis\|R_Response_to_elevated_platelet_...  B_RANKL\|K_Osteoclast_diff.  W_G13_Sign._  B_CDMAC\|K_Hepatitis_B  K_T_cell_receptor_sign.\|R_Act._of_Rac  K_Shigellosis  B_P53\|K_p53signaling_Cell  B_TGFB\|W_TGF_beta_Sign._  B_CERAMIDE  B_GCR  K_Cocaine_addiction\|K_Alcoholism  R_Chromatin_organization\|R_Chromatin_modifyin...  B_RAC1  W_Electron_Transport_Chain\|W_Oxidative_phosph...  R_Reg._of_HSF1-mediated_heat_shock_response  B_BCR\|B_CALCINEURIN\|B_FCER1\|B_GPCR\|B_TCR  R_Immunoregulatory_interact.s_between_a_Lymph...  R_G_alpha_(12/13)_sign._events  R_Metab._of_carbohydrates\|R_Glycosaminoglycan...  R_RNA_Polymerase_I,_RNA_Polymerase_III,_and_M...  K_Rheumatoid_arthritis  B_BCELLSURVIVAL  R_The_citric_acid_(TCA)_cycle_and_respiratory...  R_Toll_Like_Receptor_3_(TLR3)_Cascade\|R_TRIF-...  K_Antigen_proc._and_presentation  W_Pentose_Phosphate_\|K_Pentose_phosphate\|R_Pe...  B_PML  R_Membrane_Trafficking\|R_Golgi_to_ER_Retrogra...  B_GSK3  B_EDG1  R_HSF1_act.  R_MyD88_cascade_initiated_on_plasma_membrane\|...  B_DNAFRAGMENT  R_Inact._of_Cdc42_and_Rac  R_Detoxification_of_Reactive_Oxygen_Species  H_COMPLEMENT  R_Innate_Immune_System\|R_TRAF3-dep._IRF_act.\|...  H_UV_RESPONSE_UP  B_IL4\|W_IL-4_sign.  K_Amyotrophic_lateral_sclerosis_(ALS)  B_MTA3  W_TCR_Sign.  B_VEGF  B_EGF\|B_IGF1\|B_GLEEVEC\|B_INSULIN\|B_NGF\|B_PDGF...  R_Toll-Like_Recep._Cascades\|R_MAP_kinase_act....  K_Cytosolic_DNA-sensing  R_Translocation_of_GLUT4_to_the_plasma_membra...  K_Fc_gamma_R-mediated_phagocytosis  B_CFTR\|B_MPR\|B_PLCE  R_G1_Phase  B_CASPASE\|B_D4GDI\|SA_CASPASE_CASCADE  K_Fc_epsilon_RI_sign.\|R_GPVI-mediated_act._ca...  W_Nucleotide_Metab.  H_PI3K_AKT_MTOR_SIGNALING  K_Renal_cell_carcinoma  B_CHREBP2  W_Prostaglandin_Synthesis_and_Reg.  B_NDKDYNAMIN  B_TFF  B_RAB  R_Interferon_alpha/beta_sign.\|R_Reg._of_IFNA_...  W_Leptin_sign.\|R_Sign._by_Leptin  SA_B_CELL_RECEPTOR_COMPLEXES\|R_FCERI_mediated...  K_Neurotrophin_sign.\|R_Sign._to_p38_via_RIT_a...  H_PROTEIN_SECRETION  B_CTL\|B_TCAPOPTOSIS\|B_TCYTOTOXIC\|B_THELPER  B_HER2  K_Acute_myeloid_leukemia  R_Apoptotic_execution__phase\|R_Caspase-mediat...  H_IL2_STAT5_SIGNALING  ST_T_CELL_SIGNAL_TRANSDUCTION  K_Leishmaniasis\|K_Tuberculosis  R_Apoptosis_induced_DNA_fragmentation  W_Mismatch_repair\|K_Mismatch_repair  K_Protein_export  K_African_trypanosomiasis  W_IL-6_sign.\|R_IL-6_sign.  K_B_cell_receptor_sign.  SIG_IL4RECEPTOR_IN_B_LYPHOCYTES\|SIG_BCR_SIGNA...  K_Salmonella_infection  R_Fcgamma_receptor_(FCGR)_dep._phagocytosis\|R...  K_Estrogen_sign.  R_Toll_Like_Receptor_10_(TLR10)_Cascade\|R_IRA...  H_ANDROGEN_RESPONSE  R_Antigen_Presentation:_Folding,_assembly_and...  R_Depolymerisation_of_the_Nuclear_Lamina  K_VEGF_sign.  R_Platelet_degranulation  H_REACTIVE_OXIGEN_SPECIES  K_Carbohydrate_digestion_and_absorption\|R_Dig...  R_IRE1alpha_activates_chaperones\|R_XBP1(S)_ac...  K_Colorectal_cancer  B_HSP27  SA_MMP_CYTOKINE_CONNECTION  B_HIVNEF\|B_SODD\|B_TNFR1  B_EPO\|B_IL3\|W_IL-3_Sign.  R_Glucose_metab.\|R_Glycogen_syn.  B_BAD  W_Calcium_Reg._in_the_Cardiac_Cell\|K_Circadia...  K_HIF-1signaling  R_Sign._by_Rho_GTPases\|R_Rho_GTPase_cycle  K_Rap1signaling\|R_Sign._by_VEGF  H_P53  B_FMLP  K_Aldosterone-regulated_sodium_reabsorption  B_CREB  R_Gastrin-CREB_sign._via_PKC_and_MAPK\|R_EGFR_...  B_BLYMPHOCYTE\|K_Viral_myocarditis  K_Amoebiasis  W_IL-5_sign.  B_NFKB\|K_NF-kappa_B_sign.  B_GH  B_AGPCR\|W_Myometrial_Relaxation_and_Contracti... |
